# The Relationship between Social Support and Secondary Posttraumatic Growth among Health Care Providers Working with Trauma Victims—The Mediating Role of Cognitive Processing

**DOI:** 10.3390/ijerph19094985

**Published:** 2022-04-20

**Authors:** Piotr Jerzy Gurowiec, Nina Ogińska-Bulik, Paulina Michalska, Edyta Kędra

**Affiliations:** 1 Medical Institute, State Higher Vocation School in Glogow, 67-210 Glogow, Poland; piotr73-1973@tlen.pl (P.J.G.); edyta.kedra@wp.pl (E.K.); 2 Independent Public Clinical Hospital No. 7, Upper Silesia Medical Centre of the Medical University of Silesia in Katowice, 40-752 Katowice, Poland; 3 Department of Health Psychology, Institute of Psychology, University of Lodz, 91-433 Lodz, Poland; janina.oginska@now.uni.lodz.pl

**Keywords:** cognitive, processing, trauma, mediation, medical personnel, secondary traumatic growth, social support

## Abstract

Background: Individuals, who help trauma victims as part of their professional responsibilities, may experience positive effects of their work, occurring in the form of Secondary Posttraumatic Growth (SPTG). Its determinants include environmental factors such as social support, and individual characteristics, particularly cognitive processing of the trauma. The purpose of this study was to determine the associations between SPTG and social support and cognitive processing of trauma, also considered as a mediator, in a group of medical personnel exposed to secondary trauma. Methods: The results of 408 participants, paramedics and nurses, were analyzed. Three measurement tools were used: the Secondary Posttraumatic Growth Inventory, the Social Support Scale measuring four sources of support and the Cognitive Trauma Processing Scale to assess five cognitive coping strategies. Results: The results indicated that SPTG was positively related to social support and cognitive coping strategies. Cognitive coping strategies act as a mediator in the relationship between social support and SPTG. Conclusions: Despite their exposure to secondary traumatization, paramedics and nursing staff experience positive consequences of their work related to helping injured people. In order to promote growth after trauma, it is advisable to encourage medical representatives to use social support and primarily positive coping strategies.

## 1. Introduction

As a group, medical personnel are regularly exposed to professional stress, including traumatic stress [1,2,3,4,5]. In the process of helping people who have experienced trauma, medical personnel also become indirectly exposed to that trauma. The type of trauma includes, but is not limited to, accidents (most often traffic accidents), violence, and also serious, life-threatening illnesses [6]. The term vicarious traumatic exposure has been used to refer to indirect traumatic exposure [7]. With up to 80% of the general population experiencing a traumatic event within their lifetime, indirect exposure to trauma could be considered relatively common among health professionals [8]. As first responders, medical personnel are usually the first to provide assistance to victims. The negative consequences of indirect trauma are usually reflected in the form of Secondary Traumatic Stress (STS), also known as Secondary Traumatic Stress Disorder (STSD) [9]. Secondary traumatic stress is a disorder caused primarily by negative reactions from a person directly exposed to trauma. This coverage of secondary trauma was primarily due to the modification of the diagnostic criteria for PTSD in the DSM-IV classification, in particular the extension of the definition of a traumatic event to witness or be aware of serious injury or death to another person. Secondary traumatic stress is the onset of posttraumatic stress symptoms resulting from indirectly experienced traumatic events [10]. The symptoms of secondary traumatic stress, such as PTSD, have been grouped into four main categories: intrusion, avoidance, alternations in cognition and mood, and alternations in arousal and reactivity [6]. It is important that STS is considered a phenomenon that occurs in people who support trauma victims whose clinical picture is similar to PTSD and is not defined as a specific mental disorder in DSM V [6].

Experiencing a traumatic event does not always result in posttraumatic stress disorder or secondary traumatic stress as a reaction to the event. Sometimes the trauma does not change one’s behaviours, emotions, or cognitive strategies from pre-trauma exposition. Working with trauma victims can also be a source of positive posttraumatic changes, often labelled Secondary/Vicarious Posttraumatic Growth (SPTG/VPTG). SPTG is defined as the personal growth and meaning that can be gained through another’s trauma [11]. Much like the posttraumatic growth (PTG) experienced by traumatised victims, these positive changes relate to self-perception, relationships with others, and philosophy of life [7,12,13,14,15]. Despite the significant similarities between PTG and SPTG, some differences between these constructs can be distinguished. For example, SPTG is characterised by aspects related to professional identity, including the awareness that their work has value, as well as professional opportunities and competencies [16,17,18,19]. By contrast, these characteristics do not exist in PTG.

SPTG develops through the same process as PTG [17,20,21]. Referring to the PTG model [20,22,23], it can be assumed that the patients’ traumatic experiences represent a significant challenge to the cognitive patterns of the professionals helping them. These challenges trigger cognitive strategies to process and cope with the trauma. The authors of the model also highlight the importance of the environmental and individual human characteristics that existed prior to the trauma experience. In addition, environment in the form of social support can, directly and indirectly, contribute to the occurrence of positive traumatic changes by fostering cognitive trauma processing.

Comparative studies involving various professional groups that work with trauma victims have shown a relatively high level of SPTG among medical personnel [5,14]. Lev-Wiesel et al. [24] reported higher levels of SPTG among nurses compared to social workers. Similarly, a high degree of positive secondary traumatic changes has been shown by midwives participating in life-threatening childbirths [25,26] and by nurses employed in palliative care units [27]. Moderate SPTG severity was found among psychiatric and community nurses [28].

The positive consequences of indirect exposure to trauma depend on both internal and external factors. Internal factors include individual characteristics, of which a significant role is attributed to the cognitive processing of trauma. External factors include organisational circumstances such as workload and support received from the working environment [7,13,14,17,29,30].

### 1.1. The Role of Social Support in the Occurrence of SPTG

Social support, especially in the workplace, can not only reduce the severity of the stress experienced but can also encourage the occurrence of positive posttraumatic changes in those who help trauma victims. The role of social support is emphasised in models of PTG [23,31]. These models suggest that social support positively influences coping and adaptation to trauma, improves social resources and decreases isolation and loneliness. According to Schaefer and Moos [32], social support enhances personal resources such as character strengths, enhances social resources by promoting interpersonal relationships, and aids the development of further coping skills. Many authors indicate that sources of support, such as support received from friends, family, colleagues, or superiors are important [5,14,29]. The possibility of revealing one’s thoughts, expressing emotions or sharing feelings after contact with traumatized clients may contribute to reducing the intensity of the perceived stress and contributing to the occurrence of positive effects. However, this possibility varies, taking into account the people and sources that provide this support. Joseph [33] claimed that discussing a traumatic experience with supportive peers enhances one’s ability to process a traumatic event, adopt new perspectives, gain new insights, and ultimately achieve PTG. Manning-Jones et al. [14] emphasised that social support enables the correction of distorted cognitive patterns as a result of the trauma experienced by the victim. This suggests that social support can promote the effective management of trauma and the occurrence of SPTG both directly and also indirectly through cognitive trauma processing.

Social support has been linked to the development of SPTG in various groups of professionals working with trauma victims [7,14,34,35,36]. Furthermore, Kang et al. [29] reported that, among members of ambulance service teams, increases in SPTG occur not only after subjective and objective social support but also after the search for support. Furthermore, social support, mainly from peers, was reported to be a significant negative predictor of STS among nurses [37]. Ogińska-Bulik and Juczyński [5] also found a positive association between SPTG and support from co-workers in medical personnel (no significant links in a group of nurses). Nurses who experienced secondary trauma showed positive correlations between social support and vicarious growth after trauma in the study of Mairean [38]. In addition, individuals who are reporting posttraumatic stress symptoms also less frequently reported high levels of posttraumatic growth when experiencing high levels of social support.

### 1.2. The Role of Cognitive Trauma Processing in the Occurrence of SPTG

Secondary exposure to trauma, as with exposure to directly experienced trauma, often leads to a disturbance in one’s basic assumptions about the world. The violation of these key assumptions then makes it possible to reconstruct them, which in turn leads to positive posttraumatic changes. The way survivors cognitively process the traumatic event is a crucial factor in their likelihood of developing PTG [13,30]. The observation of positive posttraumatic changes in the victim increases the possibility of SPTG in those who help them [19]. Cognitive processing can take many different forms. One of them is the concept of Williams et al. [39] who described this issue in terms of cognitive coping strategies: positive cognitive restructuring, i.e., finding positive sides in the traumatic experience; downward comparison, i.e., interpreting the traumatic experience as one that appears less harmful than others; resolution/acceptance, i.e., making attempts accepting a traumatic event; denial, i.e., avoiding processing information related to the trauma; and regret, i.e., reacting with sadness, depression in a traumatic situation and the inability to cope.

The Cohen and Collens [17] model of SPTG shows the importance of coping efforts, both behavioural and cognitive. However, so far, few studies have been conducted to verify these assumptions, especially with regard to cognitive activity. One supporting study shows that midwives who have participated in complicated childbirths demonstrate a positive association between the challenges to key beliefs and SPTG [26]. This suggests that flexibility of cognitive patterns, expressed as readiness for change, promotes the positive effects of secondary exposure to trauma.

One study assessed positive cognitive restructuring in staff and volunteers from three domestic violence organisations [30]. Analyses showed a positive correlation with resolution and a negative correlation with regret. Furthermore, regression analyses found that four out of five cognitive processing strategies significantly contributed to SPTG. Ogińska-Bulik and Juczyński [5] showed that cognitive trauma processing is the main predictor of SPTG. All three positive coping strategies (assessed with CPOTS) were positive predictors of SPTG, i.e., resolution/acceptance, positive cognitive restructuring, and downward comparison.

It is possible that cognitive coping strategies act as a pathway from social support to SPTG, that is, it may mediate the relationship. It may be that following social support, professionals working with trauma victims engage in various, mainly positive, coping strategies to help them deal with the trauma, and in turn, these coping strategies promote SPTG.

### 1.3. Aim of the Research

This research aimed to establish links between social support and cognitive trauma processing and SPTG in medical personnel exposed to secondary trauma, taking into account the mediating role of cognitive trauma processing in the relationship between social support and SPTG. The following four sources of social support were considered: support from superiors, co-workers, family, and friends. Indicators of cognitive trauma processing were cognitive trauma coping strategies.

The adopted research model references the PTG model [20,22,23,31], which emphasises the importance of both cognitive processing of trauma and social support in the occurrence of positive posttraumatic changes. It also referred to the Cohen and Collens model [7] which emphasises the role of both the behavioural and cognitive efforts of the individual in the occurrence of positive changes after trauma.

It is predicted that both social support and positive cognitive coping strategies will be positively linked to SPTG and positive coping strategies will mediate the relationship between social support and SPTG. It is important to point out that an alternative model assuming the mediating role of social support could exist. However, cognitive processing of trauma seems to be a better mediator when citing cognitive concepts of trauma and PTG [22,23,40].

## 2. Materials and Methods

### 2.1. Type of Study

The study involved 430 medical personnel who provide medical assistance to injured patients as part of their professional duties. The study was conducted from November 2019–February 2020 in provincial emergency stations, emergency medical teams, emergency departments of several hospitals in Poland, as well as in oncology, intensive care and hospice wards. The present project has the approval of a bioethics committee of Opole Medical School. The study was cross-sectional. The questionnaires were delivered to and collected by the authors or persons trained by the authors during medical staff working hours after prior initial oral approval of the study. Moreover, the respondents completed a written consent form, which a clause stating that filling in the questionnaires was an informed consent to the study. The criterion for study inclusion was working as a nurse or paramedic and helping people who experience traumatic events, for example, those struggling with illness, i.e., heart attack, stroke, cancer or after an accident.

### 2.2. Participants

The results of 419 participants aged 19–65 years (M = 39.60, SD = 11.03) were collected. Data relating to 11 people were not included in the analysis as their questionnaires were not fully completed; they were dropped out. Among the subjects 137 (32.7%) were male and 282 (67.3%) were female. The study group included paramedics (*n* = 201), where 60.2% were men, and the nursing team (*n* = 218), where the vast majority were women (92.7%). The majority of rescuers helped people after various types of accidents, mainly traffic accidents (57.2%), the rest after injuries such as strokes, and heart attacks (42.8%). The nursing team included those working with oncology patients (87.7%) and accident victims (18.3%). The length of service of the medical personnel surveyed ranged from 1 to 43 years (M = 12.18, SD = 9.74), the number of hours per week devoted to assisting injured patients ranged from 2 to 90 (M = 38.64, SD = 15.64), and the workload, expressed as the percentage of work devoted to directly assisting patients in relation to all work performed, ranged from 2 to 100% (M = 69.11, SD = 31.89).

### 2.3. Measures

The present study employed a survey that included questions about age, type of events experienced by patients, employment history in the paramedic/nurse profession, number of hours per week spent working with patients, workload expressed by the percentage of direct assistance to patients concerning all duties performed, as well as the three standard measurement tools described below:

The Secondary Posttraumatic Growth Inventory—SPTGI, as developed by Ogińska-Bulik and Juczyński [5], is designed to measure positive changes related to exposure to indirect trauma in professionals. It contains 12 statements assessed on a 6-degree scale, from “I have not experienced this change” (0) to “I have experienced this change to a very large degree” (5). It led to identifying four factors, namely, (1) new challenges and increased professional skills; (2) an increase in spiritual experiences and a sense of responsibility for others; (3) greater self-confidence and appreciation of life, and (4) an increase in acceptance and acting for the benefit of others. Each factor is composed of three statements. The SPTG was fulfilled in relation to the events listed in the inclusion criteria for the study group. High indicators of reliability were obtained and expressed by Cronbach’s α coefficient, namely 0.90 for the whole scale and 0.71; 0.85; 0.89; 0.87 for individual factors, respectively.

Social Support Scale—Whose Support You Can Count On is part of the Psychosocial Working Conditions questionnaire [41]. The tool allows for the measurement of support received from both work-related sources, comprising superiors and co-workers, as well as outside of work, i.e., from family and friends (“To what extent can you count on someone helping you in a specific way?”). The tool consists of eight statements which participants answer on a 5-point scale from 1 (very small extent) to 5 (very large extent). The psychometric properties of the scale are satisfactory (support from superiors: α = 0.94, co-workers: α = 0.92, friends from outside work: α = 0.89, and family: α = 0.89). Cronbach’s alpha obtained for scale was 0.86.

The Cognitive Processing of Trauma Scale (CPOTS), developed by Williams, Davis, and Millsap [39], was adapted to Polish conditions by Ogińska-Bulik and Juczyński [42]. The tool consists of 17 statements (“Overall, there is more good than bad in this experience”) and measures five cognitive processing aspects: positive cognitive restructuring, resolution/acceptance, downward comparison, regret and denial. Research participants are asked to relate to every statement on a 7-degree scale, from −3 (strongly disagree) to 3 (strongly agree). The score for each scale is counted separately. The reliability of the Polish version of CPOTS was assessed using Cronbach’s *alpha* coefficient, and the result was satisfactory. The generated coefficients were: 0.84 for positive cognitive restructuring, 0.89 for downward comparison, 0.82 for resolution/acceptance, 0.56 for denial, and 0.72 for regret. The present study employed a version of the questionnaire adjusted to the investigation of people indirectly exposed to trauma.

### 2.4. Data Analysis

In order to verify the data obtained from the survey, IBM SPSS, version 25 (Armonk, NY, USA: IBM Corp.) was used. In the first step of the analysis, averages and standard deviations were calculated. Pearson correlation coefficients were used to analyse relationships between variables. Finally, mediation analysis was conducted to confirm that the mechanism underlying the occurrence of positive secondary posttraumatic changes is based on the cognitive activity undertaken by the participants. Mediation analysis was conducted using the bootstrapping procedure proposed by Preacher and Hayes [43]. The independent variable, which acts as a predictor, was social support received from superiors, co-workers, family, and friends. The dependent variable was SPTG and the mediators were cognitive coping strategies. A 95% confidence interval was assumed for the analysis.

## 3. Results

### 3.1. Descriptive Statistics

The mean result for SPTG is 32.35 (SD = 13.92). It shows that an average of 31.06% of participants reported SPTG. (The data used in the analysis come from a larger project. Some of them may be used in another article.) This result is similar to the average obtained in the studies of five groups of professionals working with trauma victims [5]. According to the SPTG criteria, a low, average, and high level of secondary growth were reported by 27.4%, 32.5%, and 40.1% of respondents, respectively.

Work-related factors did not affect severity of SPTG. Firstly there was no significant difference between the two study groups, the paramedics and the nursing teams (M = 32.13, SD = 13.67 and M = 32.54, SD = 14.16, respectively, *p* > 0.05, t = −0.29). Secondly, there was no significant difference between the type of events experienced by patients (accidents, trauma/diseases) and SPTG (M = 33.31, SD = 12.39 and M = 31.79, SD = 14.73, *p* > 0.05, *t* = 1.08). Finally, workload indicators in the form of length of service, number of working hours per week, and the percentage of the work spent providing direct assistance to victims, were not associated with SPTG (respectively: r = 0.09; r = 0.08; r = 0.08). Demographic data also failed to show any effect; neither gender (men: M = 31.34, SD = 13.09; women: M = 32.84, SD = 14.29, *p* > 0.05, *t* = −1.03), nor age (r = −0.01), were associated with SPTG.

### 3.2. Correlation Coefficients between Social Support, Cognitive Trauma Processing and SPTG

Social support and cognitive coping strategies positively correlated with SPTG and its dimensions (Table 1). It should be noted that support from family (r = 0.26, *p* < 0.01) and friends (r = 0.30, *p* < 0.001) generated slightly higher correlation coefficients than support from superiors (r = 0.20, *p* < 0.01) and co-workers (r = 0.22, *p* < 0.01). All of the cognitive coping strategies analysed, both positive and negative, were positively correlated with the level of SPTG. However, as expected the higher values of correlation coefficients were related to positive strategies, i.e., positive cognitive restructuring (r = 0.52, *p* < 0.001), resolution/acceptance (r = 0.48, *p* < 0.001), and downward comparison (r = 0.33, *p* < 0.001).

### 3.3. Mediation Analysis

**Figure 1:**
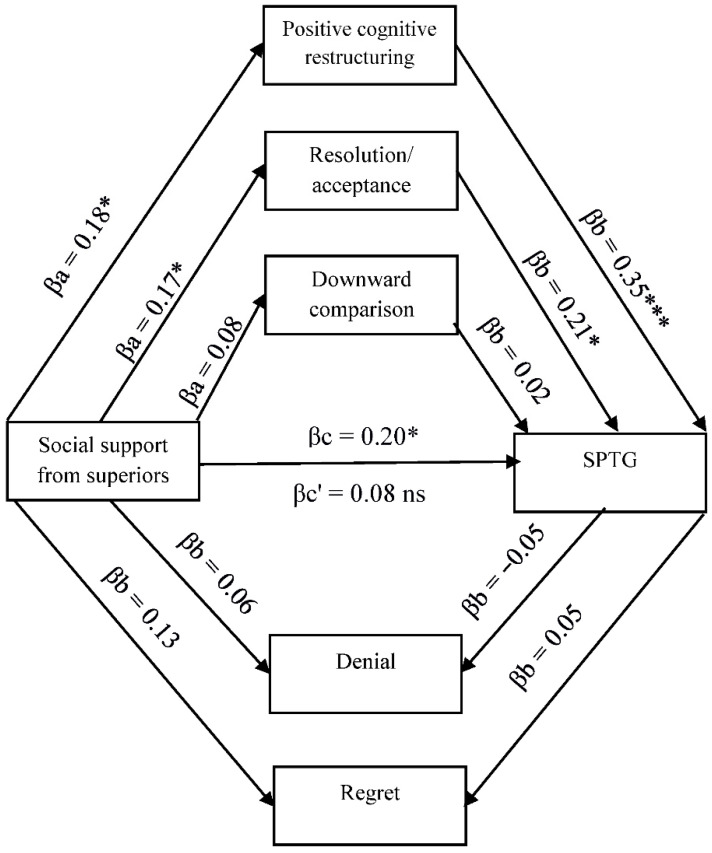
Model of relations between social support from superiors, cognitive processing of trauma and secondary posttraumatic growth. βa,b—indirect effect; βc—total effect; βc’—direct effect. * *p* < 0.05; *** *p* < 0.001.

**Figure 2:**
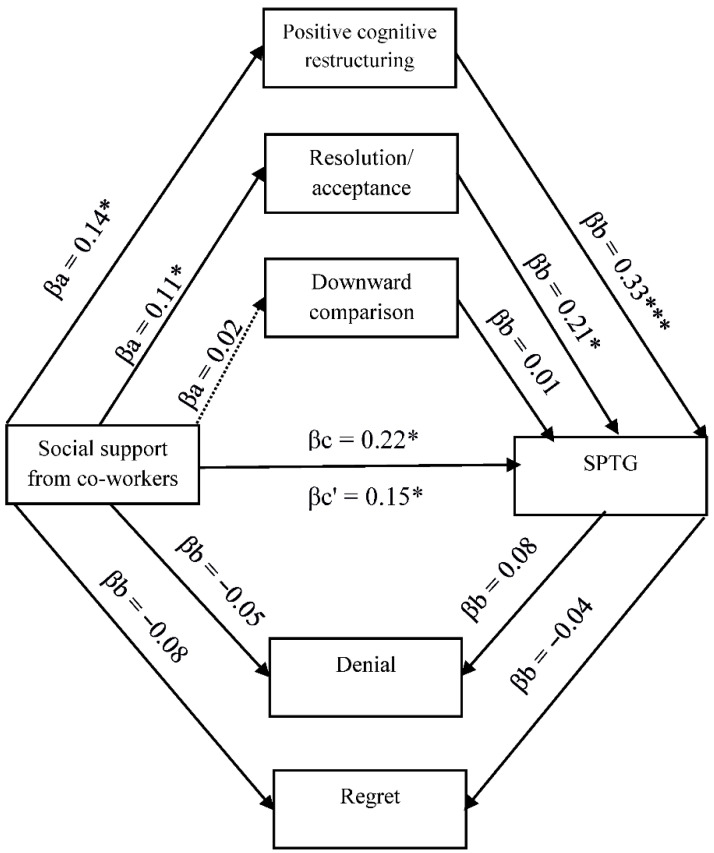
Model of relations between social support from co-workers, cognitive processing of trauma and secondary posttraumatic growth. βa,b—indirect effect; βc—total effect; βc’—direct effect. * *p* < 0.05; *** *p* < 0.001.

**Figure 3:**
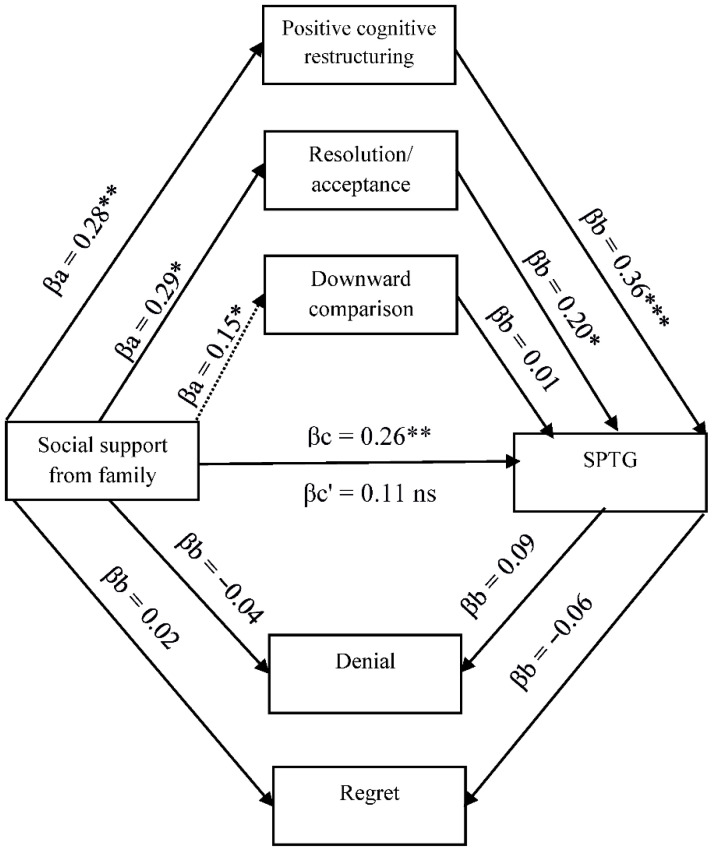
Model of relations between social support from family, cognitive processing of trauma and secondary posttraumatic growth. βa,b—indirect effect; βc—total effect; βc’—direct effect. * *p* < 0.05; ** *p* < 0.01; *** *p* < 0.001.

**Figure 4:**
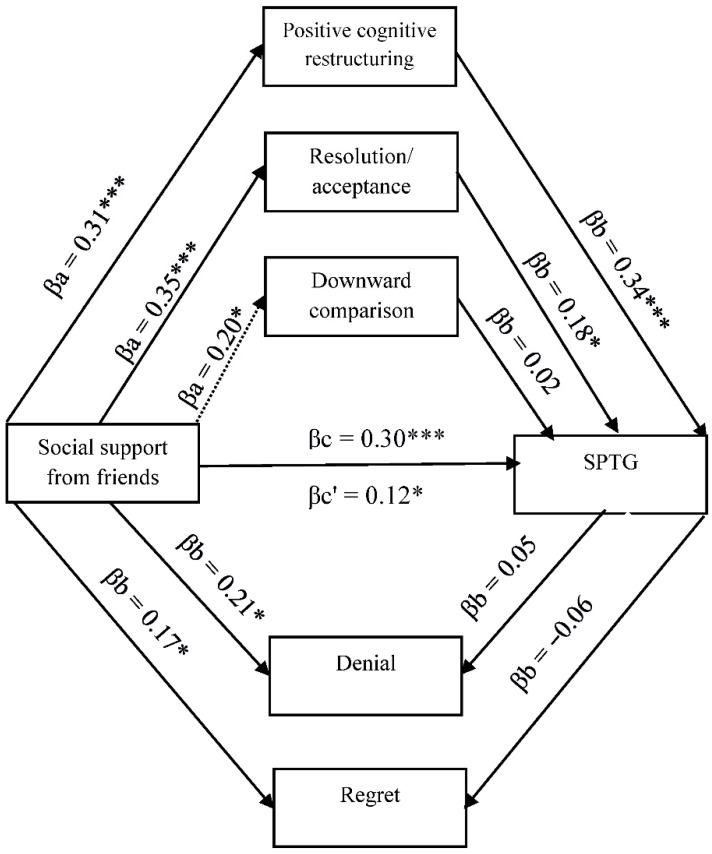
Model of relations between social support from friends, cognitive processing of trauma and secondary posttraumatic growth. βa,b—indirect effect; βc—total effect; βc’—direct effect. * *p* < 0.05; *** *p* < 0.001.

The existing relationships between SPTG, social support, and cognitive trauma processing justify the search for more complex relationships, including the mediating role of cognitive processing of trauma. Four models in mediation analysis with multiple mediators were tested (Figure 1, Figure 2, Figure 3 and Figure 4).

As can be seen in Figure 1, social support obtained from superiors is positively linked with positive cognitive restructuring and resolution/acceptance. Both variables are positive predictors of SPTG, with the coping strategy being a much stronger predictor. The introduction of mediating variables in the form of cognitive coping strategies in the relationship between social support from superiors and SPTG causes the relationship to become statistically insignificant, which indicates full mediation. Cognitive coping strategies in the form of positive cognitive restructuring and resolution/acceptance remain the only predictors of SPTG. Downward comparison, regret and denial turned out to be non-significant in mediation analysis.

Figure 2 shows the links between the support provided by co-workers, cognitive coping strategies and SPTG. Cognitive coping strategies in the form of positive cognitive restructuring and resolution/acceptance act as partial mediators between support and SPTG, reducing the strength of the relationship between the variables. This means that social support affects SPTG both directly and indirectly through positive cognitive restructuring and resolution/acceptance increasing the likelihood of positive secondary posttraumatic changes.

Figure 3 shows the relationship between family support, cognitive coping strategies and SPTG. Two coping strategies, positive cognitive restructuring and resolution/acceptance fully mediated the relationship between social support from family and SPTG.

Figure 4 shows the links between support from friends, cognitive coping strategies and SPTG. Positive cognitive restructuring and resolution/acceptance act as partial mediators in the relationship between support from friends and SPTG weakening the strength of the relationship between independent and dependent variables.

## 4. Discussion

Despite secondary trauma exposure, examined medical personnel experienced positive posttraumatic changes, manifested as vicarious growth. As many as 40% of the respondents experienced a high level of growth. The findings are in accordance with the previous studies [1,5,8].

Additionally, in line with previous studies [5,14,15,29,44,45], the current research found that social support is positively linked to SPTG. Furthermore, the correlational analysis showed that the different types of social support are not equally beneficial; social support from outside the working environment, i.e., from friends and family produced higher correlation coefficients. This is consistent with research into the type of social support [7,33,34,35,45]. Support from family and friends may reduce the impact of STS, provide helpful resources, help correct distorted perceptions, and offer a place to express reactions that may be inappropriate to share with patients. Results suggest that different forms of social support play an important role in enhancing the well-being of professionals. Overall, the findings are consistent with research that has identified social support to be one of the most vital coping strategies available to health professionals [8]. It should be noted, however, that the reported levels of social support reflect the perceived availability of support, not the support actually received.

Social support was also found to be positively linked to cognitive strategies for coping with trauma; again this was particularly true of the support received from friends, which correlates with all coping strategies, both positive and negative. This suggests that the relationship between social support and secondary posttraumatic changes depends—at least partly—on the source of support. The support received from others, and especially from friends, therefore seems to promote cognitive trauma processing.

SPTG was positively linked to all cognitive coping strategies, although less so to negative strategies. Links to positive strategies were stronger, especially positive cognitive restructuring. The results obtained are fully in line with expectations for positive strategies and correspond to the results of other studies [5,30]. It is worth noting the positive—albeit weak—links between SPTG and negative strategies, i.e., regret and denial. Such associations indicate that the display of negative emotions and feelings of responsibility for the patient’s pain and suffering, as well as denial of events, can, at least to some extent, encourage the occurrence of positive secondary posttraumatic changes. It can be assumed that living with regret for the suffering of others helps medical personnel to find answers to existential questions, including the meaning of life. This, in turn, may lead those who experience vicarious trauma to re-evaluate their own lives, to give them a new meaning, and thus to changes that are developmental in nature. These cognitive coping strategies make up the processing of trauma, and this means that they may be subject to change, possibly depending on the severity of the trauma and the passage of time. It is possible that negative strategies (regret, denial) may be accompanied by positive strategies in the form of cognitive restructuring or resolution/acceptance. However, in medical personnel, the application of denial strategies may be a form of defence against the considerable stress resulting from being confronted with the pain and suffering of patients, and may thus foster the occurrence of the positive effects of indirectly experienced trauma. It is also possible that negative coping strategies in the form of regret and denial do in fact lead to the negative effects of secondary exposure to trauma, most often in the form of symptoms of STS. These, however, can in turn act as a catalyst for positive change. This would be in line with the PTG model [20,22], which indicates that the same mechanism of cognitive trauma processing lies at the root of both negative and positive posttraumatic changes. This is also in line with the results of research conducted on people who were directly exposed to traumatic events [46]. It is important to notice that when confronted with stress, including traumatic stress, people use different coping strategies, both negative and positive (just as they experience different emotions), and these strategies are generally subject to change depending on the passage of time.

The cognitive processing of trauma, which is primarily expressed in the form of positive coping strategies, was shown to be a partial mediator in the relationship between social support from co-workers and friends, and SPTG, and a full mediator in the relationship between support from superiors and family and SPTG. This applies primarily to two positive coping strategies, cognitive restructuring and resolution/acceptance. As expected, positive strategies favour the occurrence of secondary positive posttraumatic changes more than social support, regardless of the source of social support. This means that health professionals are most likely to experience SPTG if they receive social support, both within and outside the work environment, and apply positive cognitive trauma coping strategies. Therefore, these findings indicate that support from others increases SPTG directly and indirectly through the engagement in positive cognitive processing of trauma. Previous investigations of secondary trauma have also found that cognitive strategies acted as mediators [46]. Negative cognitive coping strategies turned out to be non-significant for the relationship between social support and SPTG.

Generally, cognitive processing of trauma allows those who have experienced trauma to both, directly and indirectly, revise their assumptions about themselves and the world. It is also connected with the ability to give the experienced event sense and meaning and thus adapt to a new, changed reality. The current research, therefore, confirms that the PTG model, which highlights the importance of social support and an individual’s cognitive activity in the face of trauma, can also be applied to vicarious growth. It is also worth noting that the strength of the link between the cognitive activity of helpers and SPTG seems to be similar to that of the link between cognitive activity and PTG [46].

## 5. Limitations

The current research has certain limitations. For example, it was a cross-sectional design, which does not allow conclusions to be drawn about cause and effect relationships. In addition, the study relied entirely upon self-report data. As such there was no objective measure of the social support participants actually received. Furthermore, the results presented in the current study should not be considered generalizable to all medical staff working with trauma victims. The study did not take into account the importance of directly experienced traumatic events, either work-related or personal. This could have impacted the results since available studies reported a positive association between SPTG and personal trauma history [45,47]. When measuring social support, the focus was on sources; their types, for instance, emotional or instrumental, were not analysed. The studied group was not homogeneous; among paramedics, men were the predominant group, whereas, among nursing staff, women were the predominant group. Additionally, the search for social support can also be considered a coping strategy. Another dependence between variables that cannot be ruled out is that social support is a mediator in the relationship between cognitive trauma processing and SPTG. Some limitations may also be low internal consistency for the denial subscale.

Despite the limitations indicated, the results of this study bring new content to the scope of conditions for positive consequences of secondary exposure to trauma in medical personnel, demonstrating the importance of both social support and the cognitive processing of trauma. One significant advantage of this research is the large group of respondents, including not only nurses but also paramedics, who are less frequently studied. A particularly important aspect of this study is the use of a new measurement tool. The Secondary Posttraumatic Growth Inventory was developed specifically to evaluate positive posttraumatic changes in professionals working with trauma victims. Study on secondary trauma seems to be especially important nowadays when individuals are facing the negative effects of a COVID pandemic and a war crisis. Helping individuals may be at this time particularly vulnerable to secondary traumatization, therefore it seems so important to look for factors that may play a protective role from negative reactions, expressed in PTSD symptoms or favour the emergence of positive effects.

The analyses carried out here may provide inspiration for further research into other indicators of cognitive trauma processing, such as ruminating about experienced events or disturbances in basic beliefs. It also seems important to assess the significance of medical personnel’s personal resources when it comes to the occurrence of SPTG. We predict that the sense of self-efficacy will be particularly important, as well as the empathy and emotional bond with patients. It also seems appropriate to establish the mediation role of cognitive trauma processing in the relationship between organisational factors, such as workload or job satisfaction, as well as the characteristics of an individual’s personality and SPTG. Additional ideas for further research include longitudinal studies that make it possible to capture developments in SPTG and studies that recruit other groups of medical professionals who are exposed to secondary trauma.

## 6. Implications for Practice

Practical implications of this research include greater insight into the development of preventive programmes aimed at promoting SPTG. It would be beneficial to broaden trauma management competence to include positive cognitive strategies as well as social support. It also seems important to implement practices aimed at self-care. Self-care is an important factor that supports the ability to help professionals effectively assist others and may improve the quality of their work and personal lives [48,49]. Moreover, SPTG may be important for human functioning, probably including health and quality of life, which is an important area of public health.

## 7. Conclusions

Medical personnel, comprising paramedics and a nursing team experienced secondary exposure to trauma. Despite this, they displayed positive posttraumatic changes in the form of SPTG. The results indicated positive links between SPTG and social support and cognitive coping strategies. Cognitive coping strategies, especially those of a positive nature, act as a mediator between social support and SPTG. In order to promote posttraumatic growth, it is recommended that, first and foremost, medical personnel be empowered to use positive coping strategies. In addition, they should be encouraged to utilize all possible social support.

## Figures and Tables

**Table 1:** Correlation coefficients, mean, and standard deviations (*N* = 419).

Variables	1	2	3	4	5	6	7	8	9	10
1. SPTG total	-									
2. social support from supervisors	0.20 **	-								
3. social support from co-workers	0.22 **	0.62 ***	-							
4. social support from family	0.26 **	0.21 **	0.40 ***	-						
5. social support from friends	0.30 ***	0.47 ***	0.26 ***	0.49 ***	-					
6. positive cognitive restructuring	0.52 ***	0.19 **	0.15 **	0.28 ***	0.31 ***	-				
7. downward comparison	0.33 ***	0.08	0.02	0.15 **	0.20 **	0.59 ***	-			
8. resolution/acceptance	0.48 ***	0.17 **	0.11 *	0.29 ***	0.35 ***	0.67 ***	0.51 ***	-		
9. denial	0.23 **	0.07	−0.08	0.02	0.17 **	0.43 ***	0.63 ***	0.33 ***	-	
10. regret	0.24 **	0.14 **	−0.05	−0.04	0.21 **	0.39 ***	0.48 ***	0.30 ***	0.70 ***	-
Mean	32.35	23.44	27.95	29.80	25.78	8.66	8.14	12.20	8.61	6.42
Standard deviation	13.92	8.38	7.38	7.62	8.42	4.23	4.43	5.46	5.22	4.15

SPTG = secondary posttraumatic growth; * *p* < 0.05; ** *p* < 0.01; *** *p* < 0.001.

## Data Availability

The data that support the findings of this study are available from the corresponding author, P.M., upon request.
